# 

**Published:** 1878-09

**Authors:** 


### Books and Pamphlets Received.

Anatomy, Descriptive and Surgical, by Henry, Gray, F. R. S., fellow of the Royal College of Surgeons, and lecturer on Anatomy at St. George's Hospital Medical School, with five hundred and ninety-two engravings on wood, a new American, from the eighth and enlarged English edition. Philadelphia: Henry C. Lea. Buffalo: Theodore H. Butler. pp. 983. 1878.

On the Therapeutic Forces, by Thos. J. Mays, M. D. Philadelphia: Lindsay & Blakiston. Buffalo: T. H. Butler. pp. 143. 1878.

Fownes' Manual of Chemistry. Theoretical and practical; revised and corrected; by Henry Watts, B. A., F. R. S. Philadelphia: Henry C. Lea. Buffalo: Theodore H. Butler. pp. 1627. 1878.

Annual Reports of the Supervising Surgeon-General of the Marine Hospital Service of the United States, for the fiscal years 1876 and 1877. John M. Woodworth, M. D.

Minutes of the State Medical Society of Arkansas, at its Third Annual Session, Little Rock.

Forcible and rapid Dilitation of the Cervix Uteri, for the relief of Stricture, Chronic Endo-Cervicitis, Chronical Cervix, Flexions, Sterility, etc., by John Ball, M. D., of Brooklyn, N. Y.

Addresses delivered June 13, 1818, by B. L. Hovey, M. D., of Rochester, N. Y., before the Alumni, and Wm. C. Wey, M. D., of Elmira, N. Y., before the Graduating Class of the College of Medicine, of the Syracuse University. 32 pages.

Transactions of the Medical Society of the State of New York, for the year 1878. Truair, Smith & Bruce, printers, Syracuse. pp. 329. 1878.

Bibliotheca Medica. Robert Clarke & Co. Cincinnati.

### THE BUFFALO

### MEDICAL AND SURGICAL

Books and Pamphlets Received, ..... 80

THE BUFFALO

MEDICAL AND SURGICAL JOURNAL.

PUBLISHED MONTHLY,—Containing Original Articles, Reports of Medical Societies and Hospitals, Editorial, Reviews, Correspondence, Army News, etc., etc; including the usual variety of Medical Periodical Publications. Specimen copies sent on application to BUFFALO MEDICAL AND SURGICAL JOURNAL, J. F. MINER, M. D., 978 Main Street, BUFFALO, N. Y.

TERMS OF SUBSCRIPTION, $3 00 PER YEAR, IN ADVANCE,—All communications for publication should be sent before the fifteenth (15) of each month. or they will of necessity lie over until the next number. No change can be made in advertisements after the twenty-fifth.

Correspondence and original articles are solicited. Publication is no evidence that the views of the author are endorsed by the Journal.

